# Protocol for the economic evaluation of paraprofessional-led case management and two brief culture-informed suicide prevention interventions for American Indian youth

**DOI:** 10.21203/rs.3.rs-5450028/v1

**Published:** 2024-12-13

**Authors:** Christopher G. Kemp, Novalene Goklish, Rosemarie Suttle, Tina Minjarez, Cindy Kaytoggy, Mitchell Garcia, Robin Tessay, Heather Rock, Emily E. Haroz, Meredith Stifter, Luke A. Aldridge, Allison Barlow, Mary Cwik

**Affiliations:** Johns Hopkins University; Johns Hopkins University; Johns Hopkins University; Johns Hopkins University; Johns Hopkins University; Johns Hopkins University; Johns Hopkins University; Johns Hopkins University; Johns Hopkins University; Johns Hopkins University; Johns Hopkins University; Johns Hopkins University; Johns Hopkins University

**Keywords:** Suicide prevention, community-based, American Indian/Alaska Native, health economics

## Abstract

**Background:**

Suicide is a leading cause of death among American Indian youth, reflecting the intergenerational consequences of colonization, historical trauma, racism, and the chronic underfunding of critical health and social services in Native communities. American Indian values, spiritualities, and cultural practices promote the physical, social, and emotional health of Native people, and there is a need for community-based case management approaches and culture-informed behavioral interventions that build from this strength. Cost and cost-effectiveness estimates are critical for policymakers in Tribal communities considering investing in such services.

**Objectives:**

Our objective will be to estimate the cost and cost-effectiveness for community-based paraprofessionals to deliver three preventive services to American Indian youth (aged 10–29): case management, New Hope (a brief intervention to reduce immediate suicide risk) and Elders’ Resilience (a brief intervention incorporating Elders to increase connectedness, self-esteem, and cultural identity).

**Methods:**

We will conduct an economic evaluation as part of a Sequential Multiple Assignment Randomized Trial in a rural, reservation-based American Indian community in the Southwest. A five-year time horizon, societal perspective, and 3% discount rate will be used. An ingredients-based approach will estimate fixed program costs (e.g., intervention development, shared overhead) and variable program costs (e.g., labor and intervention delivery). Additional costs to the participants and healthcare payer will be estimated. Data collection methods will include key informant interviews, activity logs, expenditure reports and records review, direct observation, and medical chart review. Total cost estimates for each service will be divided by the respective numbers of participants reached to estimate relative cost-efficiency. Primary and secondary outcomes will be quality-adjusted life years and suicidal ideation, respectively. Incremental cost effectiveness ratios will be estimated.

**Discussion:**

We will develop much-needed estimates of the cost and cost-effectiveness of delivering community-based, paraprofessional-delivered case management and culture-informed suicide prevention interventions in a rural, reservation-based American Indian community in the Southwest. These estimates will fill a key gap for Tribal policymakers considering comparable services for their communities.

## Introduction

Suicide is a leading cause of death among American Indian and Alaska Native (AI/AN) youth, who die from suicide at two to three times the rate of the general US population of young people (1). In parts of the US Southwest, rates of suicide for AI/AN young people (aged 15–24) can be thirteen times the general US rate for the same ages (2). This inequity – which continues to grow (3) – reflects the intergenerational consequences of colonization, historical trauma, racism, the denial of tribal sovereignty, and the chronic underfunding of critical health and social services in Native communities (4).

AI/AN values, spiritualities, and cultural practices promote the physical, social, and emotional health of AI/AN people, and there is a need for community-based case management approaches and culture-informed behavioral interventions that build from this strength. Many AI/AN communities and tribal stakeholders have been leaders and innovators in this space (5–10). In 2002, the White Mountain Apache Tribe (WMAT), based on the Fort Apache Indian Reservation in Arizona, responded to a cluster of suicides among young people by establishing the Celebrating Life Suicide Surveillance and Prevention System (Celebrating Life) through tribal resolution, with support from partners at the Johns Hopkins University Center for American Indian Health (now renamed the Center for Indigenous Health, JHUCIH) (11). WMAT mandated the reporting to the Celebrating Life system of suicide ideation, attempt, binge substance use, and self-injury on the Fort Apache reservation. Case managers seek out all referred cases and offer follow-up visits to promote wellbeing and prevent future suicide events (11). In the first six years of implementing Celebrating Life, WMAT saw a 38.3% reduction in suicide deaths overall compared to the previous six years and a 23% reduction among young people aged 15–24 (12).

Seeking to further reduce the risk of youth suicide, the WMAT-JHUCIH partnership developed two brief interventions aiming to promote culture as a protective factor against suicide and to provide young people with skills to reduce risk factors, which could be integrated with the case management process already in place (13). These interventions are called ‘New Hope,’ which is focused on safety planning immediately after a suicide event, and ‘Elders’ Resilience,’ in which community Elders teach cultural knowledge, language, and values (6,13). They target key risk factors that are particularly relevant in tribal communities, including historical trauma, adverse childhood events, and alcohol and substance misuse (14,15). They also seek to leverage key protective factors and community strengths, including tribal spirituality, participation in cultural activities, social support, and connectedness to family and land (5,16–18).

Since 2019, the WMAT-JHUCIH partnership has evaluated the impact of New Hope and Elders Resilience alongside standard-of-care case management through a Sequential Multiple Assignment Randomized Trial (SMART) with a target recruitment of N=304 WMAT youth participants (15). All participants are first randomized to receive ongoing case management visits with WMAT community mental health specialists alone or in combination with the New Hope intervention and are then re-rerandomized to receive continued case management alone or in combination with the Elders Resilience intervention. The trial will determine whether New Hope, Elders Resilience, or a combination of the two interventions is more effective at reducing suicide risk and increasing resilience in these AI youth compared to case management alone.

Cost and cost-effectiveness estimates will be crucial for policymakers in Tribal communities considering investing in such services, especially given chronic under-funding and the paucity of comparable estimates developed for community-based suicide prevention interventions (19,20). Such evidence has rarely been generated in the context of AI/AN young people. Our objective will be to conduct an economic evaluation as part of this trial and to estimate the costs, cost-efficiency, and cost-effectiveness of paraprofessional-led case management and two brief culture-informed suicide prevention interventions for AI youth.

## Methods

### Parent Trial Design

The design of this trial has been described in detail previously (15). In summary, the parent study is a Sequential Multiple Assignment Randomized Trial designed to evaluate the effectiveness of New Hope (NH), Elders’ Resilience intervention (ER), Case Management (CM) and the combination of these approaches on reducing suicidal thoughts and promoting resilience among N = 304 AI youth ages 10–29 who are identified by the WMAT Celebrating Life surveillance system. The study is being implemented by WMAT and JHCIH. Participant inclusion criteria includes: 1) AI youth 10 to 29 years old; 2) residing on or near the Fort Apache Indian Reservation; 3) written consent for those over age 18, plus child assent and parent/guardian consent for youth under 18 years old; and 4) suicide ideation or suicide attempt in the past 30 days.

Potential participants are identified by Celebrating Life case managers during in-person follow-up visits that occur after individuals are referred to the Celebrating Life surveillance system. At these visits, which happen at the individual’s home or another private setting, case managers confirm information about the index event and connect them to local mental health and other social services as needed. Case managers also assess potential participants for study eligibility and obtain informed consent/assent from those who are eligible and willing.

Case managers provide all CM services and conduct all study assessments. Interventionists, who are blinded to trial arm assignment, offer the NH and ER interventions. Participants first complete a baseline assessment (CM1) and are then randomized 1:1 to either NH plus CM, or CM alone, using a blocked randomized design, stratifying participants by age and event type. Combination CM and study assessment visits are then conducted after 30 days post-enrollment (CM2) and 60 days post-enrollment (CM3). Interventionists offer the NH intervention to NH+CM arm participants between CM1 and CM2. After CM3, all participants are re-randomized, using the same blocking and 1:1 ratio, to either the ER intervention plus CM, or CM alone. Two more combination CM and study assessment visits are conducted at 90 days post-enrollment (CM4) and 180 days post-enrollment (CM5). Interventionists offer the ER intervention to ER+CM arm participants between CM3 and CM4.

Primary outcomes include suicidal ideation, as measured by the Suicide Ideation Questionnaire (SIQ) or Suicide Ideation Questionnaire – Junior (SIQ-JR), and resilience, as measured by a modified version of the Resiliency Scales for Children and Adolescents (RSEA) (21–23).

### Interventions

#### Case Management

Study assessments are administered at the start of each case management visit. Case managers then focus on building rapport with participants and making any necessary connections to behavioral or other social services, including to traditional healers or religious leaders if preferred. Participants can request additional case management visits as needed. Case managers also provide additional wellness checks as needed.

#### New Hope

NH is the result of an intensive adaptation of a brief empirically validated Emergency Department intervention (6,24). It is delivered by a trained paraprofessional community mental health provider in a single two- to four-hour visit. NH reinforces the seriousness of suicidal ideation, teaches coping skills and suicide safety planning, and aims to reduce barriers to treatment motivation, initiation, and adherence. NH includes two 20-minute videos featuring local actors, with Elders speaking in Apache, focused on the sacredness of life and the effects of suicide on the community.

#### Elders’ Resilience

ER is an adapted version of a monthly school-based curriculum developed by the WMAT-JHCIH partners that was designed to be delivered by Elders as an upstream, culturally-grounded suicide prevention intervention (13). The original curriculum is designed to be delivered in monthly 30–45 minute session within classrooms. The adapted version is a single two- to four-hour session. The curriculum covers themes like strength through prayer and tradition, respect, and community connection. ER also includes a 50-minute curriculum video, with Elders speaking in Apache, that reinforces the in-person ER curriculum.

### Perspective, Time Horizon, and Discounting

The economic evaluation will use a five-year time horizon and adopt a societal perspective. Future costs and benefits will be discounted using an annual discount rate of 3% in the base-case. Annual discount rates of 0 and 5% will be applied in sensitivity analyses to facilitate comparison with results from other economic evaluations of preventive health interventions. All costs will be estimated in $USD and inflation-adjusted to 2023 dollars.

### Identification, Measurement, and Valuation of Costs

#### Program Costs

An ingredients-based approach will be used, triangulating from several data sources to measure program cost items for each intervention including fixed costs (e.g., physical capital, curriculum development, staff training) and variable costs (e.g., staff time, fuel) (Table 1). Research costs will not be included. Annualized fixed costs and annual variable costs will be summed and divided by the annual numbers of participants reached to derive average intervention costs for each project year. Annualized fixed costs will be estimated using straight line and accelerated depreciation schedules.

In-depth interviews with program staff will be used to identify resources consumed during program implementation, to develop initial estimates for program fixed and variable costs, and to assess how close the program is to running at full capacity at different timepoints. All program staff, including administrators and case managers, will be invited to participate in these interviews.

Staff time logs, vehicle GPS trackers, and periods of time-motion observation will be used to triangulate against data from key informant interviews, estimate distances traveled to reach program participants, and allocate staff time to specific program activities. Time logs will be deployed with program staff over an intensive two-week period at mid-implementation. Staff will self-report one primary activity for each working hour of each day, and results will be used to estimate the distribution of staff time by programmatic activity. GPS trackers will be installed on program vehicles and turned on for a two-month period at mid-implementation. These will note vehicle start, stop, and idle times over the course of each day, as well as approximate addresses corresponding with each vehicle status change. In combination with program records for each day, this tracking data will be used to estimate the time and distance required to attempt to reach participants in different communities, as well as to identify any efficiencies associated with attempting to visit multiple participants in the same community on a single trip. Finally, a trained member of the costing team will conduct two separate periods of time-motion observation for up to one week each. The team member will shadow program staff members over the course of each week, note activities performed by staff, and document start and stop times.

As necessary, program expenditure reports and local data on gas and lease/rental prices will be reviewed to identify values for each of the input counts estimated through the approaches described above.

#### Participant Costs

We will assume that time spent by out-of-school participants in program activities will be borne by those participants as an opportunity cost. Accordingly, we will value that time by multiplying estimates of the average time spent in each intervention component by the local minimum wage.

#### Healthcare Payer Costs

Total medical costs for all healthcare received by study participants at the local Indian Health Service (IHS) Hospital will be estimated by reviewing electronic medical records for each participant. Data collected will include the frequency of healthcare use and the coded services used. Current Procedural Terminology (CPT) codes for the services will be converted to cost estimates using Arizona’s Medicaid fee schedule. Adjusted odds ratios representing significant differences in healthcare service usage in intervention versus control groups will be used to estimate medical cost savings across trial arms.

### Identification and Measurement of Outcomes

The primary and secondary outcomes for the cost-effectiveness analysis will be quality-adjusted life years (QALYs) and suicidal ideation, respectively. These will be measured in the parent trial as part of participant assessments conducted at baseline (CM1), 30-days post-baseline (CM2), 60-days post-baseline (CM3), 90-days post-baseline (CM4), and 180-days post-baseline (CM5).

A simple Markov model will be used to forecast QALYs and suicidal ideation over a five-year time horizon. This model will have two states – healthy and having suicidal ideation – with bilateral flow between the two states. Using a clock time of one month, an estimated background monthly incidence of suicidal ideation, and the incidence rate ratios from each trial arm, we can estimate the total number of person-years spent with suicidal ideation in each arm over five years. QALYs will be calculated by multiplying a disability weight for suicidal ideation by the estimated number of patient-years with suicidal ideation.

Given that disability weight estimates for mental health conditions are influenced by social context and can differ dramatically between communities, we opted to establish a specific disability weight estimate for the WMAT community. We used a vignette-based approach wherein Apache youth participants read and listened to narratives about characters experiencing depressive symptoms and suicidal ideation and then rated the health of those characters. This was the first study of its kind to measure disability weights for suicidal ideation among AI/AN youth (25).

As a secondary analysis, we will explore the possible intervention cost-effectiveness associated with prevention of suicide completion using a range of hypothesized reductions in the incidence of suicide given observed changes in suicidal ideation. Life-years saved through suicide prevention will be added to the estimates of QALYs under standard assumptions applying local estimates of life expectancy.

### Cost-Effectiveness Analysis

We estimate incremental cost-effectiveness ratios comparing NH vs. CM, ER vs. CM, and NH + ER vs. CM, calculated separately using QALYs and suicide ideation effectiveness estimates.

### Sensitivity Analysis

Uncertainty ranges for cost estimates will be developed that reflect any disagreement across the data collection methods used. Uncertainty ranges for effectiveness estimates will be derived from the primary trial results. A range of disability weights for suicidal ideation from the literature will be tested. Univariate (i.e., tornado plot) and probabilistic multivariate sensitivity analyses will be conducted.

## Discussion

There is little robust evidence on the effectiveness and cost-effectiveness of culturally appropriate suicide prevention interventions for young people in AI communities. A recent literature review on the cost-effectiveness of suicide and self-harm prevention interventions found six studies that offered high-quality evidence of cost-effectiveness, though none of these focused on addressing suicide in AI communities and none included culturally appropriate interventions for this population (26). This study will therefore be among the first to compare the cost and cost-effectiveness of culture-informed suicide prevention interventions for young people in AI communities in the context of a rigorous clinical trial. The results will offer useful insights for policymakers, community leaders, mental health professionals, and other stakeholders invested in reducing the high rates of suicide in this population. Such insights will be critical to sustaining effective programs over time (27).

The use of community-based paraprofessionals in this study fits with the broader movement towards task-sharing mental health, or the delegation of mental health services from specialist healthcare workers to those with fewer formal qualifications (e.g., higher educational degrees, licensure) but with appropriate training and supervision (28). The potential economic and population health benefits from such an approach are substantial, especially given the relative scarcity of specialist providers in most rural AI communities (29). Moreover, task-sharing has the potential to promote the cultural fit of health services – given that the providers are from the same community as the clients – while offering much-needed employment opportunities (28). Task-shared case management of persons who attempted suicide has already been identified as among the most effective strategies for suicide prevention, though evidence on the cost-effectiveness of culture-informed approaches is urgently needed (30,31).

Several limitations to our study design should be considered. First, data from interviews, staff time logs, and time-motion observation periods may reflect observer bias. However, we expect to offset this limitation by triangulating findings using other data sources, including vehicle GPS trackers and study records. Second, this economic evaluation will be done in the context of a randomized trial, meaning that participant engagement in intervention activities will be incentivized, and program staff will be more closely observed and supported than would be expected in routine implementation. Both will affect the external generalizability of the findings. Importantly, the generalization of the findings to other AI communities will also need to account for the diversity of these communities and their sociopolitical contexts. Because the NH and ER interventions have been designed from within WMAT culture, they may not be equally relevant or effective across all tribes and will certainly need to be adapted. Cost estimates – in particular, costs associated with travel time – would also need to be adjusted to fit the specific geographical characteristics of other communities.

This study aims to address a significant gap in the literature concerning the cost-effectiveness of culture-based suicide prevention strategies for young people in AI communities. We anticipate that results will underscore the importance and power of building from community strength and leveraging community resources in the form of task-sharing. Our findings will serve as a key resource for policymakers, community leaders, and mental health professionals in their efforts to develop and sustain effective suicide prevention programs in AI communities.

## Figures and Tables

**Table 1:** Summary of Cost Inputs and Sources by Costing Perspective

Perspective	Inputs	Data Sources (Counts)	Data Sources (Value)

Program			
• Case Management • New Hope • Elders’ Resilience • Shared	• Physical capital • Curriculum • Equipment • Training • Labor • Transportation • Incentives	• Program records • Staff interviews • Time logs • Vehicle GPS tracking • Time-motion observation	• Program records and expenditure reports • Lease and rental markets • Local gas prices

Participant			
	• Time in program activities	• Session summary forms	• Local minimum wage

Healthcare Payer			
	• Healthcare expenditure	• Chart review, IHS electronic health records	• Arizona Medicaid fee schedule

## Data Availability

Not applicable.
